# On Shapley Ratings in Brain Networks

**DOI:** 10.3389/fninf.2016.00051

**Published:** 2016-11-29

**Authors:** Marieke Musegaas, Bas J. Dietzenbacher, Peter E. M. Borm

**Affiliations:** CentER and Department of Econometrics and Operations Research, Tilburg UniversityTilburg, Netherlands

**Keywords:** brain networks, coalitional games, Shapley value

## Abstract

We consider the problem of computing the influence of a neuronal structure in a brain network. Abraham et al. (2006) computed this influence by using the Shapley value of a coalitional game corresponding to a directed network as a rating. Kötter et al. (2007) applied this rating to large-scale brain networks, in particular to the macaque visual cortex and the macaque prefrontal cortex. Our aim is to improve upon the above technique by measuring the importance of subgroups of neuronal structures in a different way. This new modeling technique not only leads to a more intuitive coalitional game, but also allows for specifying the relative influence of neuronal structures and a direct extension to a setting with missing information on the existence of certain connections.

## 1. Introduction

In this paper we consider the problem of computing the influence of a neuronal structure in a brain network. The aim of this paper is to improve upon the techniques underlying the methodology proposed by Abraham et al. (2006).

Abraham et al. (2006) considered a coalitional game in which the worth of a coalition of vertices, the neuronal structures, is defined as the number of strongly connected components in its induced subnetwork within the whole brain network. Subsequently, Abraham et al. (2006) computed the influence of a neuronal structure in a brain network by using the Shapley value of this coalitional game as a rating. Kötter et al. (2007) applied this rating to large-scale brain networks, in particular to the macaque visual cortex and the macaque prefrontal cortex based on real-life data of Young (1992) and Walker (1940).

In this paper we introduce an alternative coalitional game which in our opinion has several advantages. First of all, by satisfying superadditivity the game is more intuitive from a game theoretical point of view. Secondly, using the Shapley value of this game as an alternative rating it allows to directly specifying relative influence of neuronal structures. We apply our alternative rating model to the brain networks considered by Kötter et al. (2007) and, generally speaking, our results corroborate the findings of Kötter et al. (2007). Finally, a third advantage of the alternative approach is related to missing information on possible connections in a brain network. As this feature is a common problem, as argued by Kötter and Stephan (2003), we illustrate how our alternative approach allows for a direct incorporation of probabilistic considerations regarding missing information on the existence of certain connections.

## 2. Shapley ratings in brain networks

A brain network is a *directed graph* (*N, A*) where *N* is a set of vertices, representing a set of neuronal structures, and *A* is a set of arcs, representing the connections between the neuronal structures. Let $\bar{A}$ denote all ordered pairs (*i, j*) of vertices in *N* for which there exists a directed path from *i* to *j* in (*N, A*). A graph (*N, A*) is called *strongly connected* if for every two vertices *i* and *j* in *N* there is a directed path from *i* to *j* and from *j* to *i* in (*N, A*), i.e., if $\bar{A}$ contains all ordered pairs in *N*. The *induced subgraph* (*S, A*[*S*]) is a graph where a subset *S* ⊆ *N* is the set of vertices and *A*[*S*] is the set of arcs consisting of any arc in *A* whose starting and end point are both in *S*. A *strongly connected component* is a maximal induced subgraph which is strongly connected, i.e., there is no other strongly connected subgraph containing this strongly connected component. Let SCC(*N, A*) denote the number of strongly connected components in graph (*N, A*).

**Example 2.1** Consider the brain network (*N, A*) with *N* = {1, 2, 3, 4} illustrated below.^1^

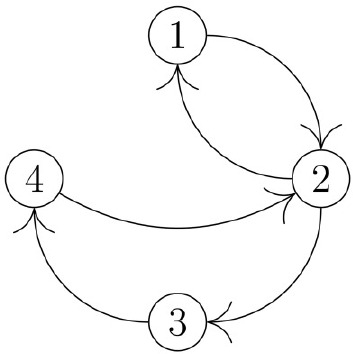



Note that (*N, A*) is strongly connected because for every vertex in the graph there exists a directed path to every other vertex. However, the subgraph induced by {1, 2, 3} is not strongly connected and we have

$$\bar{A\left[\left\{1,2,3\right\}\right]}=\left\{\left(1,2\right),\left(1,3\right),\left(2,1\right),\left(2,3\right)\right\}.$$

Note that SCC({1, 2, 3}, *A*[{1, 2, 3}]) = 2 because the subgraph induced by {1, 2, 3} consists of two strongly connected components: the subgraphs induced by {1, 2} and {3}.     △

A *coalitional game* is a pair (*N, v*) where *N* denotes a non-empty, finite set of players and *v* is a function which assigns a number to each subset *S* ⊆ *N* (also called a coalition). By convention, *v*(∅) = 0. Abraham et al. (2006) introduced a coalitional game (*N, w*^*A*^) corresponding to a brain network (*N, A*) defined by

$$w^{A}\left(S\right)=\text{SCC}\left(S,A\left[S\right]\right),$$

for all *S* ⊆ *N*. Hence, the worth of a coalition in *w*^*A*^ is defined by the number of strongly connected components in its induced subgraph.

Alternatively, we define the *brain network game* (*N, v*^*A*^) corresponding to (*N, A*) by

$$v^{A}\left(S\right)=|\bar{A\left[S\right]}|,$$

for all *S* ⊆ *N*. Hence, the worth of a coalition *S* in *v*^*A*^ is defined by the number of ordered pairs (*i, j*) of vertices in *S* for which there exists a directed path from *i* to *j* in (*S, A*[*S*]).

A basic property for coalitional games is superadditivity. A coalitional game is called called *superadditive* if breaking up a coalition into parts does not pay, i.e.,

v(S∪T)≥v(S)+v(T),

for all *S, T* ⊆ *N* with *S* ∩ *T* = ∅. From a game theoretical perspective it is desirable that coalitional games satisfy this basic property since it provides a clear incentive for cooperation in the grand coalition and thus provides a motivation to focus on fairly allocating the worth of the grand coalition. Unfortunately, this property is not satisfied by the coalitional game (*N, w*^*A*^). This is illustrated in the following example.

**Example 2.2** Reconsider the brain network (*N, A*) presented in Example 2.1. The worth of every coalition in the games (*N, w*^*A*^) and (*N, v*^*A*^) is presented below.

**Table:** 

*S*	{1}	{2}	{3}	{4}	{1, 2}	{1, 3}	{1, 4}	{2, 3}	{2, 4}	{3, 4}	{1, 2, 3}	{1, 2, 4}	{1, 3, 4}	{2, 3, 4}	{1, 2, 3, 4}
*w*^*A*^(*S*)	1	1	1	1	1	2	2	2	2	2	2	2	3	1	1
*v*^*A*^(*S*)	0	0	0	0	2	0	0	1	1	1	4	4	1	6	12

Note that (*N, w*^*A*^) is not superadditive since, e.g.,

$$w^{A}\left(\left\{1,2\right\}\right)+w^{A}\left(\left\{3,4\right\}\right)=3>1=w^{A}\left(\left\{1,2,3,4\right\}\right).$$

It is readily checked that (*N, v*^*A*^) is superadditive.     △

In contrast to the coalitional game (*N, w*^*A*^), we show in the following proposition that the brain network game (*N, v*^*A*^) does satisfy superadditivity.

**Proposition 2.1**. *Let* (*N, A*) *be a brain network. Then, the brain network game* (*N*, *v*^*A*^) *is superadditive*.

*Proof*. Let *S, T* ⊆ *N* with *S* ∩ *T* = ∅. Since *S* and *T* are disjoint, we also have $\bar{A\left[S\right]}\cap \bar{A\left[T\right]}=\emptyset$. Therefore, $\left|\bar{A\left[S\right]}|+|\bar{A\left[T\right]}|=|\bar{A\left[S\right]}\cup \bar{A\left[T\right]}\right|$ and thus for proving *v*^*A*^(*S*) + *v*^*A*^(*T*) ≤ *v*^*A*^(*S* ∪ *T*) it is sufficient to show that

$$\begin{array}{l} \bar{A\left[S\right]}\cup \bar{A\left[T\right]}\subseteq \bar{A\left[S\cup T\right]}. \end{array}$$

For showing this, let $\left(i,j\right)\in \bar{A\left[S\right]}\cup \bar{A\left[T\right]}$, i.e., there is either a directed path from *i* to *j* and from *j* to *i* in *G*[*S*] or in *G*[*T*]. Then, there is also a directed path from *i* to *j* and from *j* to *i* in *G*[*S* ∪ *T*] and thus $\left(i,j\right)\in \bar{A\left[S\cup T\right]}$.     □

The *Shapley value* [cf. Shapley (1953)] of a coalitional game (*N, v*) is for all *i* ∈ *N* defined by

$$\Phi_{i}\left(v\right)=\underset{S\subseteq N\backslash \left\{i\right\}}{\sum }p_{S}\left(v\left(S\cup \left\{i\right\}\right)-v\left(S\right)\right),$$

where $p_{S}=\frac{|S|!\left(|N|-|S|-1\right)!}{|N|!}$. Hence, the Shapley value looks at the marginal contributions of a player to all possible coalitions. The weight *p*_*S*_ is such that all marginal contributions are weighted adequately to obtain an efficient allocation of the worth of the grand coalition.

In the context of coalitional games corresponding to brain networks, the Shapley value can be interpreted as a measure for the influence of a neuronal structure. Abraham et al. (2006) considered the Shapley value Φ(*w*^*A*^) as a rating for the neuronal structures in a brain network. Similarly, we consider the Shapley value Φ(*v*^*A*^) as a rating.

**Example 2.3** Reconsider the coalitional games (*N, w*^*A*^) and (*N, v*^*A*^) of Example 2.2. The Shapley rating Φ(*w*^*A*^) is given by^2^

$$\begin{array}{l} \Phi \left(w^{A}\right)=\left(\frac{1}{2},-\frac{1}{6},\frac{1}{3},\frac{1}{3}\right), \end{array}$$

while the Shapley rating Φ(*v*^*A*^) is given by

$$\Phi \left(v^{A}\right)=\left(2\frac{1}{6},4\frac{1}{6},2\frac{5}{6},2\frac{5}{6}\right),$$

both determining a ranking (2, 3, 4, 1) or (2, 4, 3, 1) (there is a tie for the second highest ranking). We note that a lower Shapley rating in *w*^*A*^ indicates a higher influence in a brain network. On the contrary, a higher Shapley rating in *v*^*A*^ indicates a higher influence.

Since a Shapley rating in *w*^*A*^ can be negative, as is the case in this example, it is not possible to determine the relative influence of two vertices on the basis of Φ(*w*^*A*^). On the other hand, a Shapley rating in *v*^*A*^ can not be negative by definition because of superadditivity. Therefore, using Φ(*v*^*A*^), we can say that the influence of vertex 2 in the brain network (*N, A*) is almost twice as large as the influence of vertex 1.      △

A common problem in the analysis of brain networks is the fact that it is not known whether some specific connections (arcs) are present or not [cf. Kötter and Stephan (2003)]. Using a certain probabilistic knowledge about these unknown connections, this lack of information can readily be incorporated in the brain network game.

We assume that each possible arc (*i, j*) is present with probability *p*_*ij*_ ∈ [0, 1]. Clearly, for each present arc we set *p*_*ij*_ = 1 and for each absent arc we set *p*_*ij*_ = 0. All probabilities are summarized into a vector *p*. Given such a vector *p*, we define the *stochastic brain network game* (*N, v*^*p*^) in which the worth of a coalition equals the expected (in the probabilistic sense) number of ordered pairs for which there exists a directed path in its induced subgraph. Without providing the exact mathematical formulations the following example illustrates how to explicitly determine the coalitional values in a stochastic brain network game.

**Example 2.4** Reconsider the brain network presented in Example 2.1. Only now suppose that the arcs (1, 4) and (3, 1) are present with probability *p*_14_ and *p*_31_, respectively. The complete corresponding vector *p* can be found below.

**Table:** 

(*i, j*)	(1, 2)	(1, 3)	(1, 4)	(2, 1)	(2, 3)	(2, 4)	(3, 1)	(3, 2)	(3, 4)	(4, 1)	(4, 2)	(4, 3)
*p*_*ij*_	1	0	*p*_14_	1	1	0	*p*_31_	0	1	0	1	0

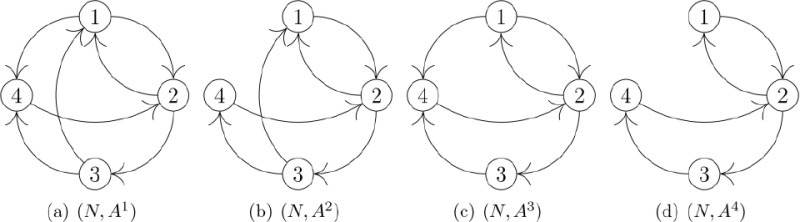



In total there are four possible brain networks. These different brain networks are illustrated above and the corresponding probabilities for those networks are *p*_14_*p*_31_, (1 − *p*_14_)*p*_31_, *p*_14_(1 − *p*_31_) and (1 − *p*_14_)(1 − *p*_31_) for (a), (b), (c), and (d), respectively.

The expected number of ordered pairs for which there exists a directed path in the induced subgraph of coalition {1, 3, 4} is computed by taking the following weighted average

$$\begin{array}{l} v^{p}\left(\left\{1,3,4\right\}\right)=p_{14}p_{31}\cdot v^{A^{1}}\left(\left\{1,3,4\right\}\right)+\left(1-p_{14}\right)p_{31}\cdot v^{A^{2}}\left(\left\{1,3,4\right\}\right)\\ \;+\;p_{14}\left(1-p_{31}\right)\cdot v^{A^{3}}\left(\left\{1,3,4\right\}\right)\\ \;+\;\left(1-p_{14}\right)\left(1-p_{31}\right)\cdot v^{A^{4}}\left(\left\{1,3,4\right\}\right)\\ \;=p_{14}p_{31}\cdot 3+\;\left(1-p_{14}\right)p_{31}\cdot 2\;+p_{14}\left(1-p_{31}\right)\cdot 2\\ \;+\;\left(1-p_{14}\right)\left(1-p_{31}\right)\cdot 1\\ \;=1+p_{14}+p_{31}. \end{array}$$

The worth of every coalition is presented below.

**Table:** 

*S*	{1}	{2}	{3}	{4}	{1, 2}	{1, 3}	{1, 4}	{2, 3}	{2, 4}	{3, 4}	{1, 2, 3}	{1, 2, 4}	{1, 3, 4}	{2, 3, 4}	{1, 2, 3, 4}
*v*^*p*^(*S*)	0	0	0	0	2	*p*_31_	*p*_14_	1	1	1	4+2*p*_31_	4+2*p*_14_	1+*p*_14_+*p*_31_	6	12

The Shapley rating of the game (*N, v*^*p*^) is given by

$$\begin{array}{l} \Phi_{1}\left(v^{p}\right)=2\frac{1}{6}+\frac{1}{3}p_{14}+\frac{1}{3}p_{31},\\ \Phi_{2}\left(v^{p}\right)=4\frac{1}{6}-\frac{1}{6}p_{14}-\frac{1}{6}p_{31},\\ \Phi_{3}\left(v^{p}\right)=2\frac{5}{6}-\frac{1}{2}p_{14}+\frac{1}{3}p_{31},\\ \Phi_{4}\left(v^{p}\right)=2\frac{5}{6}+\frac{1}{3}p_{14}-\frac{1}{2}p_{31}. \end{array}$$

For example, if $p_{14}=\frac{1}{2}$ and $p_{31}=\frac{1}{3}$, then

$$\Phi \left(v^{p}\right)=\left(2\frac{16}{36},4\frac{1}{36},2\frac{25}{36},2\frac{30}{36}\right),$$

with corresponding ranking (2, 4, 3, 1).      △

## 3. Results and discussion

In this section we apply the Shapley rating based on the brain network game (*N, v*^*A*^) to the two large-scale brain networks considered by Kötter et al. (2007) and we compare the results.

The first large-scale brain network is the macaque visual cortex with thirty neuronal structures as illustrated in Figure 1 of Kötter et al. (2007) [cf. Young (1992), based on data compiled by Felleman and van Essen (1991)]. The five brain regions with the highest ranking obtained by means of the Shapley value of the coalitional games (*N, w*^*A*^) and (*N, v*^*A*^) can be found below in (a) and (b), respectively.

**Table:** 

**(a) Top 5 of Φ(*w*^*A*^)**				**(b) Top 5 of Φ(*v*^*A*^)**	
**Ranking**	**Brain region**			**Ranking**	**Brain region**
1.	V4			1.	V4
2.	FEF			2.	FEF
3.	46			3.	Vp
4.	V2			4.	V2
5.	Vp			5.	46

Note that both ratings agree on the top 5; only with respect to the positions 3 and 5 there are some minor differences.

**Figure A1:**
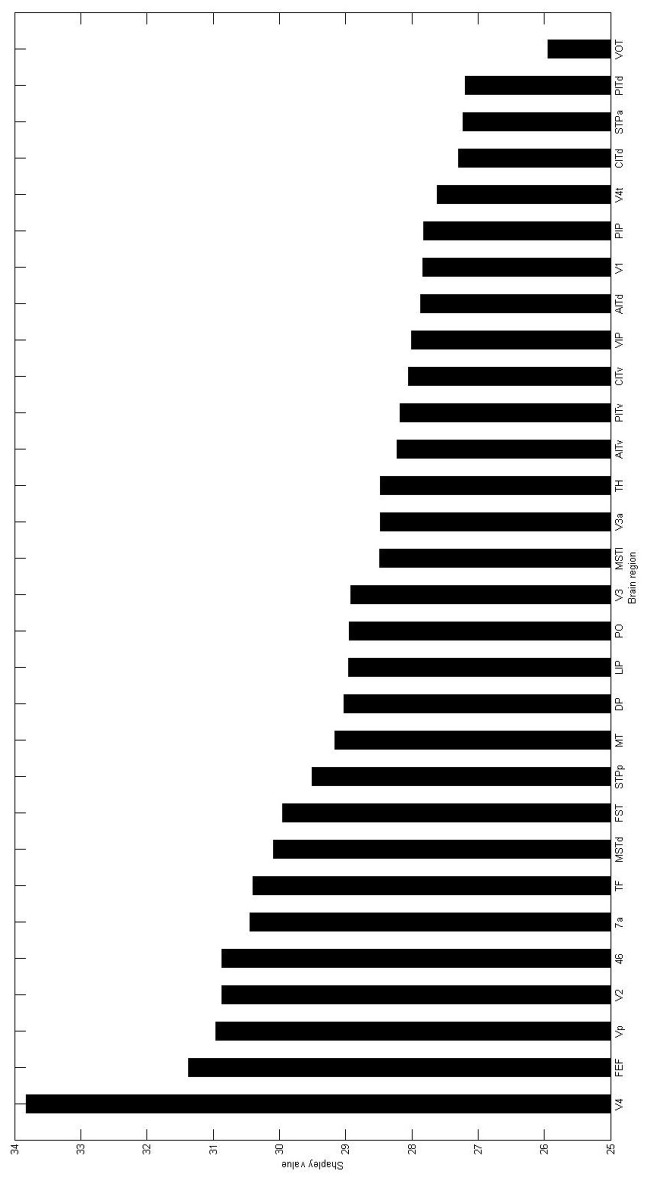
**Shapley rating of the macaque visual cortex**.

The entire Shapley rating Φ(*v*^*A*^) of the macaque visual cortex can be found in Figure A1 in the appendix. Correspondingly, we can roughly divide the brain regions in five classes based on the relative difference with the brain region with the highest Shapley rating. We consider the following five classes based on the differences in terms of percentage: 0–5%, 5–10%, 10–15%, 15–20%, 20% and higher. The first class consists of the single brain region V4 with the highest Shapley rating. The second class consists of the brain regions FEF to TF as ordered in Figure A1 that differ 5–10% with *V*4. The brain regions in the third class are MSTd to V3, in the fourth class we have MSTI to PITd and in the fifth class we have the single brain region VOT with a relative influence which is 23% lower than that of V4.

The second large-scale brain network is the macaque prefrontal cortex with twelve neuronal structures as illustrated in Figure 3A of Kötter et al. (2007) [cf. Walker (1940)]. In this case there is a lack of information about the presence or absence of nine connections. To get some insight, Kötter et al. (2007) considered two extreme cases. First, they assume that connections with unknown presence are absent. Second, they assume that those connections are present. For both extreme cases the Shapley ratings are calculated separately. Our stochastic brain network game provides a way to incorporate lack of information into one Shapley rating on the basis of probabilistic information. For simplicity, we assume that each connection with unknown presence is absent with probability $\frac{1}{2}$. Note that, in case more information would become available, more adequate probabilities can be readily inserted. Having the complete vector *p* of arc probabilities, one readily computes the corresponding stochastic brain network game (*N, v*^*p*^) and the corresponding Shapley rating Φ(*v*^*p*^). The ranking based on the Shapley rating Φ(*v*^*p*^) can be found below.

**Table:** 

**Ranking**	**Brain region**
1.	9
2.	24
3.	12
4.	10
5.	46
6.	25
7.	11
8.	8B
9.	13
10.	8A
11.	45
12.	14

## Author contributions

MM is the first author and the corresponding author. MM was present at all processes: the early research process, the programming process and the writing process. BD and PB contributed to the early research process and later on to the process of commenting on the work written by MM.

### Conflict of interest statement

The authors declare that the research was conducted in the absence of any commercial or financial relationships that could be construed as a potential conflict of interest.

## Footnotes

^1^This instance of a brain network is also used in Example 1 in Section 3.1 of Moretti (2013).

^2^Because of a mistake in the worth of *w*^*A*^({1, 2, 3}), the Shapley value is incorrectly stated by Moretti (2013).
